# Supplementary material for: Using Microarrays to Facilitate Positional Cloning: Identification of Tomosyn as an Inhibitor of Neurosecretion
Source: PLoS Genet. 2005 Jul 25;1(1):e2. doi: 10.1371/journal.pgen.0010002 (PMC1183521; doi:10.1371/journal.pgen.0010002)
Supplement: Figure S1 — Target sequences were downloaded (http://www.affymetrix.com) and aligned to unc-43 CaMKII isoform H. Since a majority of genes on the C. elegans GeneChip are represented by one probeset, unc-43 represents an atypica1 case. To explain this, it is useful to consider the history and design of the C. elegans GeneChip (see http://www.affymetrix.com/support/technical/datasheets/celegans_drosophila_datasheet.pdf). The targets for this GeneChip were designed by Affymetrix based on more than 18,800 Sanger Center predicted transcripts from the December 2000 genome sequence, as well as 2,300 3' EST clusters and 300 GenBank mRNAs (Release 121). Despite efforts to eliminate redundancy, there is not a strict one-to-one correspondence between the current set of genes and the probesets on the GeneChip. Our analysis indicates that 13% of the genes on the GeneChip are represented by more than one probe. Even so, having unc-43 CaMKII represented by seven probesets is an unusual situation. However, only two of these probesets (193459_s_at and 193463_s_at) are annotated as corresponding to the unc-43 CaMKII mRNA transcript according to annotations of the C. elegans GeneChip provided by Affymetrix in the March 28, 2003, update (downloadable at http://www.affymetrix.com). During our examination of the eight candidate probesets that showed decreased expression and were on Chromosome 4, we discovered five additional probesets that corresponded to unc-43 CaMKII. Four of these probesets (172058_x_at, 175820_s_at, 175821_s_at, and 175824_s_at) were based on GenBank sequences, and one (187759_s_at) was based on a predicted open reading frame, Y43C5B.1, that was part of the genome as of December 2000 but has since been shown to correspond to the 5′ UTR of unc-43 CaMKII. These additional probesets provided a serendipitous blind control, since we were not aware of their existence until they appeared on our candidate list from the hybridization. There is also an additional (eighth) probeset (173423 [file pgen.0010002.sg001.pdf]

Supplemental Figure 1

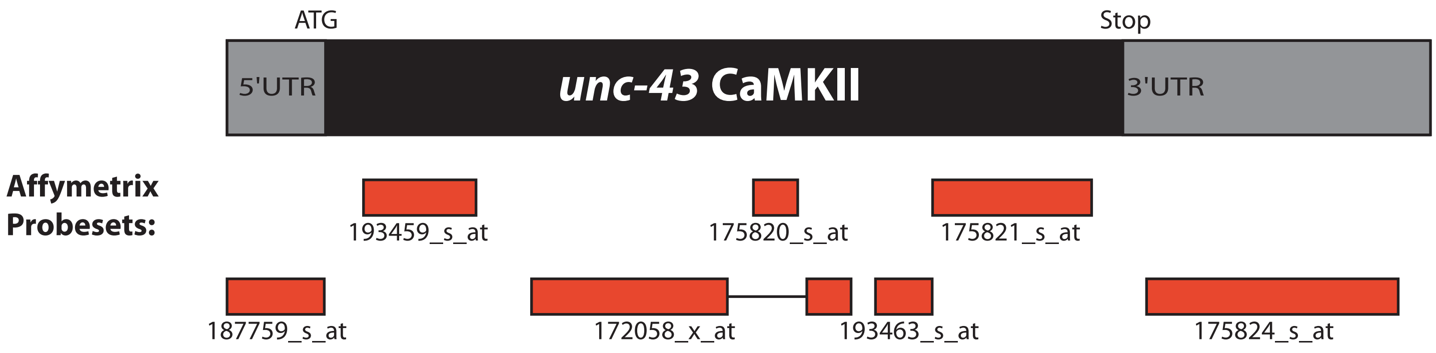

Figure S1. Probeset alignments to CaMKII *unc-43* isoform H (K11E8.1h). Target sequences were downloaded (www.affymetrix.com) and aligned to CaMKII *unc-43* isoform H. Since a majority of genes on the *C.elegans* GeneChip are represented by one probesets, *unc-43* represents an atypica1 case. To explain this, it is useful to consider the history and design of the *C.elegans* GeneChip (see www.affymetrix.com/support/technical/datasheets/celegans_drosophila_datasheet.pdf). The targets for this GeneChip were selected by Affymetrix based on over 18,800 predicted genes and splice forms in the December 2000 genome sequence, as well as 2300 clusters of EST from the Sanger Center and 300 Genbank mRNAs (release 121). Despite efforts to eliminate redundancy, there is not a strict one-to-one correspondence between the current set of genes and the probsets on the GeneChip. Our analysis indicates that 13% of the genes on the GeneChip are represented by more than one probe. Even so, having CaMKII *unc-43* represented by seven probesets is an unusual situation. However, only two of these probesets (193459_s_at and 193463_s_at) are annotated as corresponding to the CaMKII *unc-43* mRNA transcript according to annotations of the *C. elegans* GeneChip provided by Affymetrix in the March, 28 2003 update (downloadable at www.affymetrix.com). During our examination of the eight candidate probesets that were down and on chromosome IV, we discovered the five additional probesets that corresponded to CaMKII *unc-43*. Four of these probesets (172058_x_at, 175820_s_at, 175821_s_at and 175824_s_at) were based on Genbank sequences and one (187759_s_at) was based on a predicted open reading frame, Y43C5B.1, which was part of the genome as of 12/00, but has since been shown to corresponds to the 5'UTR of CaMKII *unc-43*. These additional probesets provided a serendipitous blind control, since we were not aware of their existence until they appeared on our candidate list from the hybridization.

There is also an additional (eighth) probeset (173423_at) described in the Affymetrix annotation as corresponding to CaMKII *unc-43*. However, based on sequence alignments to the current gene model, 173423_at appears to correspond to intronic sequence between exons 11 and 12. As would be expected this probeset shows no detectable expression and thus was not considered in our analysis of the CaMKII *unc-43* mutant.
