# Supplementary material for: Using Microarrays to Facilitate Positional Cloning: Identification of Tomosyn as an Inhibitor of Neurosecretion
Source: PLoS Genet. 2005 Jul 25;1(1):e2. doi: 10.1371/journal.pgen.0010002 (PMC1183521; doi:10.1371/journal.pgen.0010002)
Supplement: Figure S2 — Sequences of human and mouse tomosyn were downloaded from Ensembl (http://www.ensembl.org). The multiple sequence alignment was performed using T-Coffee (http://www.ch.embnet.org/software/TCoffee.html). Alignment output was produced using GeneDoc (http://www.psc.edu/biomed/genedoc). (104 KB PDF) [file pgen.0010002.sg002.pdf]

Supplemental Figure 2

```
                         *        20         *        40         *        60         *
TOM-1     : MDRAKKKFASAIDGLRSLHNRT--------------EINVDEKIQSEHCRFTRVVRHGLPEDPRCFAYDPVQRLIAI :  63
hTomosyn  : --MRKFNIRKVLDGLTAGSSSASQQQQQQ-HPPGNREPEIQETLQSEHFQLCKTVRHGFPYQPSALAFDPVQKILAV :  74
rTomosynS : --MRKFNIRKVLDGLTAGSSSASQQQQQQQHPPGNREPEIQETLQSEHFQLCKTVRHGFPYQPSALAFDPVQKILAV :  75

            80         *       100         *       120         *       140         *
TOM-1     : GTGRGHIRIIGDAGVDYLLKHESGEPVLHMQFLVNEGGLITSCGTDSIHLWNYRQKTAEIVHTVQLSKESVSCIHLP : 140
hTomosyn  : GTQTGALRLFGRPGVECYCQHDSGAAVIQLQFLINEGALVSALADDTLHLWNLRQKRPAILHSLKFCRERVTFCHLP : 151
rTomosynS : GTQTGALRLFGRPGVECYCQHDSGAAVIQLQFLINEGALVSALADDTLHLWNLRQKRPAVLHSLKFCRERVTFCHLP : 152

               160         *       180         *       200         *       220         *
TOM-1     : VAGKWLFIGTDKGNVYFLCLNSFLLSPYVINWNKAIDLSCRVHPGPVRQLAVSPAENTKLLIVYDKGIVVQWNLGTR : 217
hTomosyn  : FQSKWLYVGTERGNIHIVNVESFTLSGYVIMWNKAIELSSKSHPGPVVHISDNPMDEGKLLIGFESGTVVLWDLKSK : 228
rTomosynS : FQSKWLYVGTERGNIHIVNVESFTLSGYVIMWNKAIELSSKSHPGPVVHISDNPMDEGKLLIGFESGTVVLWDLKSK : 229

                 240         *       260         *       280         *       300
TOM-1     : EVD-RYPLDPPIKSVNWHFDGRQILTGNVDGSISLYNHKKSTEAIQRTTPHGSG--------PCRPIQQVEWKHMSE : 285
hTomosyn  : KADYRYTYDEAIHSVAWHHEGKQFICSHSDGTLTIWNVRSPAKPVQTITPHGKQLKDGKKPEPCKPILKVEFKTTRS : 305
rTomosynS : KADYRYTYDEAIHSVAWHHEGKQFICSHSDGTLTIWNVRSPTKPVQTITPHGKQLKDGKKPEPCKPILKVEFKTTRS : 306

             *       320         *       340         *       360         *       380
TOM-1     : TESIIMFSGGMPTDDGLPMPALTILKGGKSATVLEMDWPIIQFIPLNQNIWNSIPQCPHSVAVLLKHDFMVLDLNQN : 362
hTomosyn  : GEPFIILSGGLSYDTVGRRPCLTVMH-GKSTAVLEMDYSIVDFLTLCETPYPNDFQEPYAVVVLLEKDLVLIDLAQN : 381
rTomosynS : GEPFIILSGGLSYDTVGRRPCLTVMH-GKSTAVLEMDYSIVDFLTLCETPYPNDFQEPYAVVVLLEKDLVLIDLAQN : 382

                 *       400         *       420         *       440         *       460
TOM-1     : GHPVIESPHAMNIHESPVTCMAYYSDCPLDLIGALTLVGTKQRNKEFSARSWPVTGGLGRECATGHQELVVTGHKDG : 439
hTomosyn  : GYPIFENPYPLSIHESPVTCCEYFADCPVDLIPALYSVGARQKRQGYSKKEWPINGGNWGLGAQSYPEIIITGHADG : 458
rTomosynS : GYPIFENPYPLSIHESPVTCCEYFADCPVDLIPALYSVGARQKRQGYSKKEWPINGGNWGLGAQSYPEIIITGHADG : 459

                 *       480         *       500         *       520         *       54
TOM-1     : SLKFWQETGEHLQILYKLKSSSHFERL---EEMETS---DKVSHAVKYIELCLESRQLLVAGISGQVTLFRFTKQE- : 509
hTomosyn  : SVKFWDASAITLQVLYKLKTSKVFEKSRNKDDRPNTDIVDEDPYAIQIISWCPESRMLCIAGVSAHVIIYRFSKQEV : 535
rTomosynS : SIKFWDASAITLQVLYKLKTSKVFEKSRNKDDRQNTDIVDEDPYAIQIISWCPESRMLCIAGVSAHVIIYRFSKQEV : 536

            0         *       560         *       580         *       600         *
TOM-1     : ICGTIAVVNIPLLGNLSSNLPNNDFDKSPVPNGPGKEIRRQRKIVSRESTNSLDTSDSGDERIVPFKVRGAPVKRPP : 586
hTomosyn  : ITEVIPMLEVRLLYEINDVETPEGEQPPPLPTPVGGSNPQPIPPQSHPSTSS-SSSDGLRDNVPCLKVKNSPLKQSP : 611
rTomosynS : VTEVIPMLEVRLLYEINDVETPEGEQPPPLSTPVGSSTSQPIPPQSHPSTSS-SSSDGLRDNVPCLKRVLPSTARSN : 612

              620         *       640         *       660         *       680         *
TOM-1     : GFVPELACFVPWHSHAKV-DQITTICLNSSYGIIAIGTSSGLALVDTTQCALIYSWTTNELYGSDPTPAIQLSMQLS : 662
hTomosyn  : GYQTELVIQLVWVGGEPPQQITSLAVNSSYGL-VVFGNCNGIAMVDYLQKAVLLNLGTIELYGSN-DPYRREPRSPR : 686
rTomosynS : TSRTQDS-DFYILGNLVLFPVSRYSCMTTYDLTVVFGNSNGIAMVDYLQKAVLLNLSTIELYGSN-DPYRREPRSPR : 687

                700         *       720         *       740         *       760         *
TOM-1     : DAASPLESPIS-------------------LEAPKRIISEQKQRSFDAR---RPQLFKTQSVVENGSLSRGDSQRES : 717
hTomosyn  : KSRQPSGAGLCDISEGTVVPEDRCKSPTS------------------------------------AKMSRKLSLPTD : 727
rTomosynS : KSRQPSGAGLCDITEGTVVPEDRCKSPTSGSSSPHNSDDEQKVNNFIEKVKTQSRKFSKMVASDLAKMSRKLSLPTD : 764
```

Supplemental Figure 2 (continued)

```
                    780         *       800         *       820         *       840
TOM-1      : PSSGRQLNTSSPSTSSQSLERSMSTQPQESITSLSFIHSYSKKNDSKMSPCLWVGTSAGASLALNLILP---EDRFT :  791
hTomosyn   : LKPDLDVKDNSFSRSRSSSVTSIDKESREAISALHFCETFTRKTDSSPSPCLWVGTTLGTVLVIALNLPPGGEQRLL :  804
rTomosynS  : LKPDLDVKDNSFSRSRSSSVTSIDKESREAISALHFCETFTRKADSSPSPCLWVGTTVGTAFVITLNLPLGPEQRLL :  841

               *       860         *       880         *       900         *       920
TOM-1      : STIVVAPSGTVVRLKGQVISQSFMDNTFCIISPASESYKEAAKEQSATSPDRSLLNRVNTKASLAPQYSSSIDSNDE :  868
hTomosyn   : QPVIVSPSGTILRLKGAILRMAFLDTTGCLIPPAYEPWREHNVPEEKDEKEKLKKRR---PVSVSPSSSQEISEN-- :  876
rTomosynS  : QPVIVSPSGTILRLKGAILRMAFLDAAGCLMPPAYEPWTEHNVPEEKDEKEKLKKRR---PVSVSPSSSQEISEN-- :  913

                  *       940         *       960         *       980         *      1000
TOM-1      : ISQILIVAAENEVKVVALPTFSQLYVQKFDEIPLV-KATPTHIRGYPCLMCLSAAGQIIMLSLPSLRILQTSTIFPH :  944
hTomosyn   : --QYAVICSEKQAKVISLPTQNCAYKQNITETSFVLRGDIVALSNSICLACFCANGHIMTFSLPSLRPLLDVYYLPL :  951
rTomosynS  : --QYAVICSEKQAKVISLPTQNCAYKQNITETSFVLRGDIVALSNSVCLACFCANGHIMTFSLPSLRPLLDVYYLPL :  988

                     *      1020         *      1040         *      1060         *        10
TOM-1      : SVEIDDPLCQKTAFSDHGLGVYMASQTEMEKYTMCSEIAEQTGESLGELFVPCEMPEQPKNNSFLKGVSSIFGGTPR : 1021
hTomosyn   : T---NMRIARTFCFTNNGQALYLVSPTEIQRLTYSQETCENLQEMLGELFTPVETPEAP-NRGFFKG---LFGGGAQ : 1021
rTomosynS  : T---NMRIARTFCFANSGQALYLVSPTEIQRLTYSQETCENLQEMLGELFTPVETPEAP-NRGFFKG---LFGGGAQ : 1058

             80         *      1100         *      1120         *      1140         *
TOM-1      : NDPNDIDAILSENMGVKNSSGVNPMRSVARTIPGPS-VQMDRAQAGGVSAGQAAAMALQNLNERTEKLNATVDATEN : 1097
hTomosyn   : S--------LDREELFGESSSGKASRSLAQHIPGPGGIEGVKGAASGVVGELARARLA--LDERGQKLGDLEERTAA : 1088
rTomosynS  : S--------LDREELFGESSSGKASRSLAQHIPGPGGIEGVKGAASGVVGELARARLA--LDERGQKLSDLEERTAA : 1125
SNARE-helix:

              1160         *      1180
TOM-1      : LKNNAMSLSSRTGKLVEKYEKKKWYNF : 1124
hTomosyn   : MLSSAESFSKHAHEIMLKYKDKKWYQF : 1115
rTomosynS  : MMSSADSFSKHAHEMMLKYKDKKWYQF : 1152
SNARE-helix:
```

Figure S2. Alignment of TOM-1 to human and rat Tomosyn. Sequences of human and mouse tomosyn were downloaded from Ensembl (www.ensembl.org/), accession numbers ENSP00000179882 and ENSRNOP00000018806 respectively. The multiple sequence alignment was performed using T-Coffee (http://www.ch.embnet.org/software/TCoffee.html). Alignment output was produced using GeneDoc (www.psc.edu/biomed/genedoc).
