# Supplementary material for: Using Microarrays to Facilitate Positional Cloning: Identification of Tomosyn as an Inhibitor of Neurosecretion
Source: PLoS Genet. 2005 Jul 25;1(1):e2. doi: 10.1371/journal.pgen.0010002 (PMC1183521; doi:10.1371/journal.pgen.0010002)
Supplement: Table S1 — (51 KB PDF) [file pgen.0010002.st001.pdf]

Supplemental Table 1

Gene	Missense	Nonsense	Total	% NMD	Source
aap-1		1	1	100%	Wormbase
acy-1	17		17	0%	Wormbase
aex-1		7	7	100%	Doi, 2002
air-1	2		2	0%	Wormbase
akt-1	2		2	0%	Wormbase
anc-1		1	1	100%	Wormbase
aph-1	2	5	7	71%	Wormbase
bam-2	2	1	3	33%	Wormbase
bas-1		3	3	100%	Wormbase
bre-2	1	1	2	50%	Wormbase
bre-4	1	1	2	50%	Wormbase
bre-5	1	1	2	50%	Wormbase
cam-1	1	2	3	67%	Wormbase
cat-2		1	1	100%	Wormbase
cat-4	1		1	0%	Wormbase
cdc-25.1	2		2	0%	Wormbase
cdf-1		1	1	100%	Wormbase
cdk-4	1	2	3	67%	Wormbase
cdk-7	1		1	0%	Wormbase
cdl-1	1	1	2	50%	Wormbase
ced-1	8	4	12	33%	Wormbase
ced-10	2		2	0%	Wormbase
ced-12		7	7	100%	Wormbase
ced-2		3	3	100%	Wormbase
ced-3	1		1	0%	Wormbase
ceh-36	1	3	4	75%	Wormbase
ceh-37	1		1	0%	Wormbase
cfi-1		1	1	100%	Wormbase
che-1	5	6	11	55%	Wormbase
che-3		1	1	100%	Wormbase
chk-2	1	1	2	50%	Wormbase
cog-1	1		1	0%	Wormbase
crt-1		2	2	100%	Wormbase
ctb-1	1		1	0%	Wormbase
cup-5	2	1	3	33%	Wormbase
cyd-1		1	1	100%	Wormbase
cye-1		2	2	100%	Wormbase
cyk-3		2	2	100%	Wormbase
cyk-4	7		7	0%	Wormbase
daf-2	12		12	0%	Wormbase
dcr-1		2	2	100%	Wormbase
deg-3	11		11	0%	Wormbase
dep-1		1	1	100%	Wormbase
dgk-1	1	2	3	67%	Nurrish et al 99
dim-1		2	2	100%	Wormbase
dpy-11	7	4	11	36%	Wormbase
dpy-17	1	2	3	67%	Wormbase
dpy-19	3	1	4	25%	Wormbase
dpy-22		3	3	100%	Wormbase
dyb-1		1	1	100%	Wormbase
dyn-1	1		1	0%	Wormbase
eat-16	2	1	3	33%	Hajdu-Cronin et al, 1999
eat-18	1	1	2	50%	Wormbase
eat-2	6	1	7	14%	Wormbase
eff-1	2		2	0%	Wormbase

Supplemental Table 1

Gene	Missense	Nonsense	Total	% NMD	Source
egl-13	2	1	3	33%	Wormbase
egl-15	12	7	19	37%	Wormbase
egl-18		3	3	100%	Wormbase
egl-20	3		3	0%	Wormbase
egl-21	1		1	0%	Wormbase
egl-3	4		4	0%	Wormbase
egl-4	2	5	7	71%	Wormbase
egl-46		1	1	100%	Wormbase
egl-8		1	1	100%	Wormbase
ego-1	4	1	5	20%	Wormbase
elo-1	2		2	0%	Wormbase
eor-1	1	1	2	50%	Wormbase
eor-2		4	4	100%	Wormbase
epi-1	4	2	6	33%	Wormbase
eri-1		1	1	100%	Wormbase
evl-14		1	1	100%	Wormbase
evl-20		1	1	100%	Wormbase
exc-5		1	1	100%	Wormbase
exp-1	4		4	0%	Wormbase
fat-1		2	2	100%	Wormbase
fat-2	1		1	0%	Wormbase
fat-3	2		2	0%	Wormbase
fat-4	3	1	4	25%	Wormbase
fbf-2		1	1	100%	Wormbase
fog-1	13	5	18	28%	Wormbase
fsn-1		1	1	100%	Wormbase
fzr-1	1		1	0%	Wormbase
fzy-1	1		1	0%	Wormbase
gad-1	1		1	0%	Wormbase
gem-4	3		3	0%	Wormbase
gld-2	1		1	0%	Wormbase
glp-1	3		3	0%	Wormbase
goa-1	1	1	2	50%	Robatzek and Thomas, 2000
gon-2	3		3	0%	Wormbase
gon-4		4	4	100%	Wormbase
gpa-16	1		1	0%	Wormbase
gpb-2	6	2	8	25%	Robatzek, 2001
hda-1	1		1	0%	Wormbase
hen-1	1		1	0%	Wormbase
hid-1	2	1	3	33%	Wormbase
him-10	1		1	0%	Wormbase
hlh-2	2		2	0%	Wormbase
hlh-8	1		1	0%	Wormbase
hse-5	1		1	0%	Wormbase
hst-6	1	1	2	50%	Wormbase
hus-1	1		1	0%	Wormbase
inx-3		1	1	100%	Wormbase
inx-6	1		1	0%	Wormbase
isp-1	1		1	0%	Wormbase
kin-29	1		1	0%	Wormbase
ksr-2		1	1	100%	Wormbase
let-23	9	1	10	10%	Aroian, 1994
let-268	3		3	0%	Wormbase
let-363		3	3	100%	Wormbase
let-413	1	1	2	50%	Wormbase

Supplemental Table 1

Gene	Missense	Nonsense	Total	% NMD	Source
let-418		3	3	100%	Wormbase
let-502	9		9	0%	Wormbase
let-767	1		1	0%	Wormbase
let-99		3	3	100%	Wormbase
lin-1	1		1	0%	Wormbase
lin-10	1	9	10	90%	Whitfield, 98
lin-13	3	3	6	50%	Wormbase
lin-14		2	2	100%	Wormbase
lin-23	1		1	0%	Wormbase
lin-26	3	1	4	25%	Wormbase
lin-31	3	5	8	63%	Wormbase
lin-45	9	3	12	25%	Wormbase
lin-48	3		3	0%	Wormbase
lin-49	1	2	3	67%	Wormbase
lin-5	2	2	4	50%	Wormbase
lin-52	1	1	2	50%	Wormbase
lin-7		3	3	100%	Simske, 96
lin-9	1	2	3	67%	Wormbase
lip-1	2		2	0%	Wormbase
lon-1	7	3	10	30%	Wormbase
lon-3		3	3	100%	Wormbase
lrs-2		1	1	100%	Wormbase
mab-23	1		1	0%	Wormbase
mat-2	7		7	0%	Wormbase
mat-3	12		12	0%	Wormbase
mbk-2		3	3	100%	Wormbase
mec-6	1	3	4	75%	Wormbase
mes-1	2	3	5	60%	Wormbase
mes-3	4	1	5	20%	Wormbase
mes-4		1	1	100%	Wormbase
mig-23	2	1	3	33%	Wormbase
mre-11	1		1	0%	Wormbase
mtm-6	1	1	2	50%	Wormbase
mtm-9		1	1	100%	Wormbase
mua-6	1	1	2	50%	Wormbase
mut-14	1		1	0%	Wormbase
nmy-1	2	1	3	33%	Wormbase
npr-1	4	1	5	20%	Wormbase
nsy-1		3	3	100%	Wormbase
nuc-1		3	3	100%	Wormbase
odr-2	2	1	3	33%	Wormbase
odr-7	2		2	0%	Wormbase
oma-1	4	2	6	33%	Wormbase
oma-2	1		1	0%	Wormbase
ooc-3		1	1	100%	Wormbase
ooc-5	1	2	3	67%	Wormbase
osm-5	1	3	4	75%	Wormbase
pag-3	1	1	2	50%	Wormbase
par-5	3		3	0%	Wormbase
pat-4		1	1	100%	Wormbase
pat-6		2	2	100%	Wormbase
pen-2		6	6	100%	Wormbase
pop-1	2		2	0%	Wormbase
pqe-1	2	4	6	67%	Wormbase
pry-1		2	2	100%	Wormbase

Supplemental Table 1

Gene	Missense	Nonsense	Total	% NMD	Source
rde-4		1	1	100%	Wormbase
ref-1	1		1	0%	Wormbase
rfl-1	1		1	0%	Wormbase
ric-8	2		2	0%	Wormbase
rme-1	3	1	4	25%	Wormbase
rpm-1		4	4	100%	Wormbase
sad-1	3	1	4	25%	Wormbase
scc-3		1	1	100%	Wormbase
sec-23		1	1	100%	Wormbase
sel-12		2	2	100%	Wormbase
sel-7	2	2	4	50%	Wormbase
sel-8		1	1	100%	Wormbase
sem-4	1	2	3	67%	Wormbase
sem-5		1	1	100%	Wormbase
sid-1	7	2	9	22%	Wormbase
slo-1	3	4	7	57%	Wang, 2001
sma-9		2	2	100%	Wormbase
smg-5		3	3	100%	Wormbase
smu-1	1	2	3	67%	Wormbase
snb-1	3	1	4	25%	Nonet, 1998
soc-1		12	12	100%	Wormbase
sol-1	1	1	2	50%	Wormbase
sos-1	3		3	0%	Wormbase
spd-5	1		1	0%	Wormbase
spe-39		3	3	100%	Wormbase
spe-6	18	1	19	5%	Wormbase
spn-4	2		2	0%	Wormbase
spr-1		1	1	100%	Wormbase
spr-4		1	1	100%	Wormbase
spr-5	2	2	4	50%	Wormbase
sqt-2	2		2	0%	Wormbase
sqv-1	3		3	0%	Wormbase
sqv-2	1	1	2	50%	Wormbase
sqv-4	2		2	0%	Wormbase
sqv-6		1	1	100%	Wormbase
sup-10	2	2	4	50%	Wormbase
sup-9	49	16	65	25%	Wormbase
syd-1		2	2	100%	Wormbase
syp-2		1	1	100%	Wormbase
tax-6	1	1	2	50%	Kuhara, 2002
tbb-2	3		3	0%	Wormbase
tbg-1	1		1	0%	Wormbase
tpa-1	26	17	43	40%	Wormbase
ttx-1	1		1	0%	Wormbase
ttx-3	1	1	2	50%	Wormbase
twk-18	3		3	0%	Wormbase
ubc-18	1		1	0%	Wormbase
unc-10		5	5	100%	Koushika, 2001
unc-103	3	1	4	25%	Wormbase
unc-119		2	2	100%	Wormbase
unc-16	1	2	3	67%	Wormbase
unc-17	12		12	0%	Wormbase
unc-18	2	3	5	60%	Sassa, 99
unc-2	2	5	7	71%	Wormbase
unc-26	4	4	8	50%	Wormbase

Supplemental Table 1

Gene	Missense	Nonsense	Total	% NMD	Source
unc-27		3	3	100%	Wormbase
unc-37	2	1	3	33%	Wormbase
unc-38	2		2	0%	Wormbase
unc-43	8	3	11	27%	Reiner et al, 99
unc-5	1	6	7	86%	Wormbase
unc-59	2		2	0%	Wormbase
unc-61		2	2	100%	Wormbase
unc-62		1	1	100%	Wormbase
unc-71	14	6	20	30%	Wormbase
unc-78	3	1	4	25%	Wormbase
vab-10	2		2	0%	Wormbase
vab-9		1	1	100%	Wormbase
vps-34	2	3	5	60%	Wormbase
wee-1.3	9	2	11	18%	Wormbase
zag-1		3	3	100%	Wormbase
zen-4	5		5	0%	Wormbase
zyg-1	2	1	3	33%	Wormbase
zyg-12	2		2	0%	Wormbase
zyg-8	7	2	9	22%	Wormbase
**Total**	**559**	**384**	**943**	**41%**	
